# A review on poly(3-hydroxybutyrate-*co*-3-hydroxyhexanoate) [P(3HB-*co*-3HHx)] and genetic modifications that affect its production

**DOI:** 10.3389/fbioe.2022.1057067

**Published:** 2022-12-05

**Authors:** Hui Jia Tang, Soon Zher Neoh, Kumar Sudesh

**Affiliations:** Ecobiomaterial Research Laboratory, School of Biological Sciences, Universiti Sains Malaysia, Pulau Pinang, Malaysia

**Keywords:** polyhydroxyalkanoate, poly(3-hydroxybutyrate-*co*-3-hydroxyhexanoate) [P(3HB-*co*-3HHx)], metabolic pathway, PHA synthase, engineering of PhaCs

## Abstract

Polyhydroxyalkanoates (PHAs) have garnered global attention to replace petroleum-based plastics in certain applications due to their biodegradability and sustainability. Among the different types of PHAs, poly(3-hydroxybutyrate-*co*-3-hydroxyhexanoate) [P(3HB-*co*-3HHx)] copolymer has similar properties to commodity plastics, making them a suitable candidate to replace certain types of single-use plastics, medical devices, and packaging materials. The degradation rate of P(3HB-*co*-3HHx) is faster than the commercial petroleum-based plastics which take a very long time to be degraded, causing harmful pollution to both land and marine ecosystem. The biodegradability of the P(3HB-*co*-3HHx) is also dependent on its 3HHx molar composition which in turn influences the crystallinity of the material. Various metabolic pathways like the common PHA biosynthesis pathway, which involves *phaA, phaB,* and *phaC*, β-oxidation, and fatty acids *de novo* synthesis are used by bacteria to produce PHA from different carbon sources like fatty acids and sugars, respectively. There are various factors affecting the 3HHx molar composition of P(3HB-*co*-3HHx), like PhaCs, the engineering of PhaCs, and the metabolic engineering of strains. It is crucial to control the 3HHx molar composition in the P(3HB-*co*-3HHx) as it will affect its properties and applications in different fields.

## Introduction

Petroleum-based plastics are used in our daily life due to their characteristics like being cheap, light, resistant to chemicals, and convenient (Loo and Sudesh, 2007). Due to those desirable characteristics, they are applied in many sectors, such as packaging, medical equipment, household utensils, construction, etc. However, it was estimated that more than a million tons of plastic waste are being disposed into land and marine environment yearly due to improper disposal (Alabi et al., 2019; Masry et al., 2021). When exposed to sunlight and wind, plastic wastes are broken down into microplastics, causing toxic effects on aquatic life and human health (Bajt, 2021).

**FIGURE 1:**
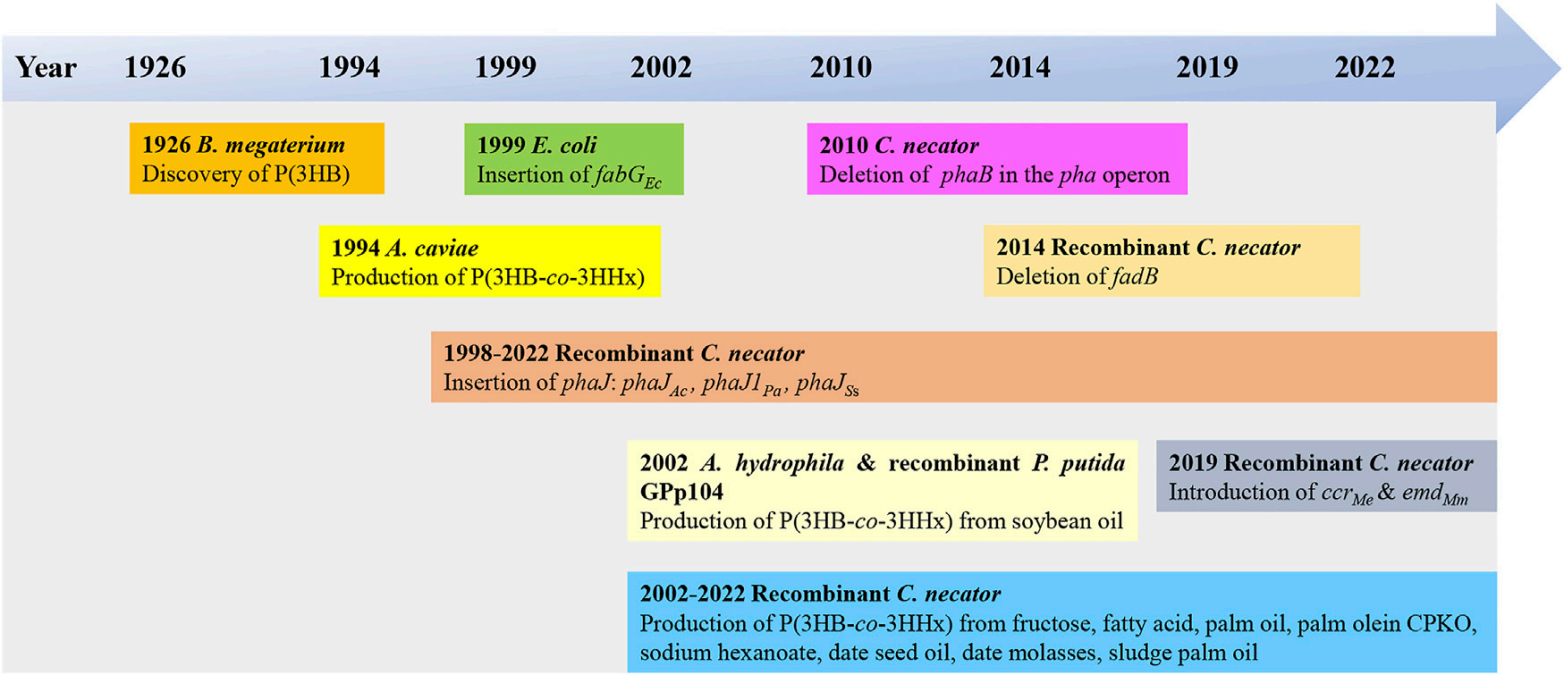
Timeline of the P(3HB-*co*-3HHx) developments (Lemoigne, 1926; Shimamura et al., 1994; Fukui et al., 1998; Asrar et al., 2002; Fukui et al., 2002; Tsuge et al., 2004; Loo et al., 2005; Budde, 2010; Wong et al., 2012; Insomphun et al., 2014; Volova et al., 2016; Murugan et al., 2017; Purama et al., 2018; Thinagaran and Sudesh, 2019; Zhang et al., 2019; Tan et al., 2020; Tan et al., 2022)

**FIGURE 2:**
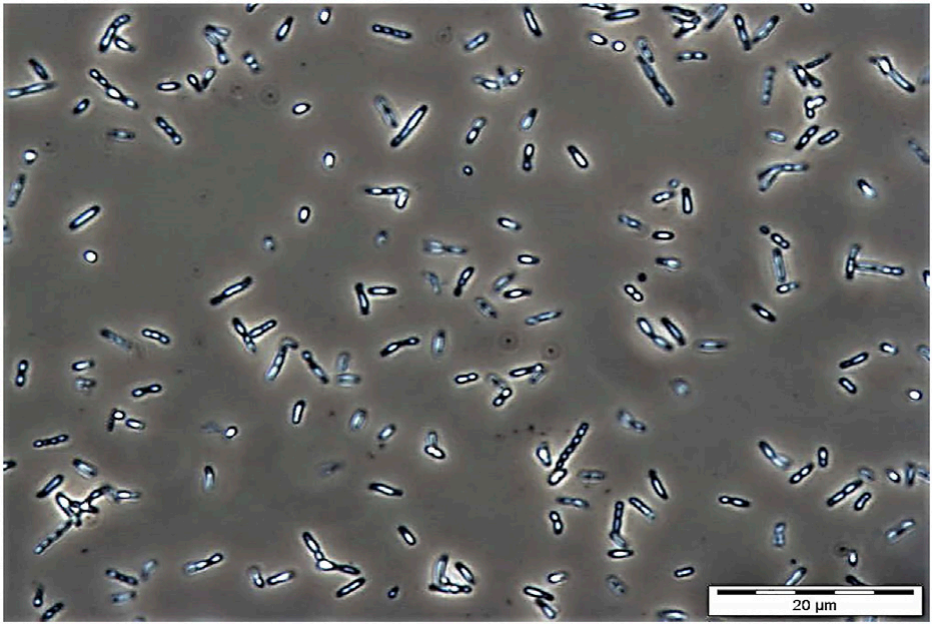
Phase contrast microscopic image of PHA granules in *C. necator* mutant Re2058 harboring plasmid pHT1 with *phaC* from *Chromobacterium* sp. USM2 (*phaC*_*Cs*_) containing P(3HB-*co*-3 mol% 3HHx) copolymer after cultivating in minimal medium at 30°C, 200 rpm for 48 h with 0.54 g/L of urea as nitrogen source and supplemented with crude palm kernel oil (CPKO). Magnification: 1000 ×. The PHA content produced was 60 wt%.

The use of bio-based plastics as one of the alternatives to replace single-use petroleum-based plastics may be one of the options for solving this issue. There are several potential bio-based plastics, such as polyhydroxyalkanoates (PHAs), polylactides (PLA), polysaccharides, etc. Among the bio-based plastics, PHAs have been reported to have interesting properties like biodegradable, thermoplastic, renewable, and can be tailored to fit various applications attracted both academic and industrial (Sudesh and Iwata, 2008). PHA was first discovered by Lemoigne in *Bacillus megaterium* (Lemoigne, 1926). The timeline of the P(3HB-*co*-3HHx) developments is shown in Figure 1. PHAs are bio-based polymers produced by a wide range of microorganisms under deprived nutrients and excessive carbon sources (Anderson and Dawes, 1990; Fukui and Doi, 1998). They are deposited as intracellular granules and function as energy or carbon reservoir (Anderson and Dawes, 1990; Sudesh et al., 2000). Figure 2 shows the PHA granules in cell cytoplasm synthesized by *Cupriavidus necator* transformant under phase contrast light microscope.

PHAs can be divided into three groups, short-chain-length PHA (SCL-PHA) of 3–5 carbon units, medium-chain-length PHA (MCL-PHA) of 6–14 carbon units, and a mixture of both SCL- and MCL-PHA of 3–14 carbon units (Sudesh et al., 2000). Examples of SCL monomeric units are 3-hydroxybutyrate (3HB) and 3-hydroxyvalerate (3HV), while MCL monomeric units are 3-hydroxyhexanoate (3HHx) and 3-hydroxydodecanoate (3HD) (Sudesh, 2012). Example of the mixture of SCL- and MCL-PHA is poly [(*R*)-3-hydroxybutyrate-*co*-(*R*)-3-hydroxyhexanoate] P(3HB-*co*-3HHx). The monomers incorporated into PHA polymers will affect the thermal and physical properties of the PHAs produced.

Poly(3-hydroxybutyrate) [P(3HB)] homopolymer is brittle, stiff, has high crystallinity, and low elongation at break, causing it to be limited in many applications (Yu, 2007). This drawback of PHA homopolymers can be overcome by copolymerizing P(3HB) with MCL monomers for better properties (Noda et al., 2005). For instance, the incorporation of MCL-PHA monomer like 3HHx into P(3HB) results in P(3HB-*co*-3HHx) copolymer, which has elastomeric properties such as high elasticity, low crystallinity, and high elongation at break. The flexibility of this copolymer can be varied depending on the 3HHx molar compositions (Asrar et al., 2002). P(3HB-*co*-3HHx) with 17 mol% 3HHx molar fraction was also reported to have similar properties as low-density polyethylene (LDPE) (Doi, 1990; Doi et al., 1995).

PHA synthase (PhaC) is the key enzyme in PHA production since it is responsible for catalyzing the polymerization of PHA monomers into PHA polymers. In addition, it also determines the monomer composition integrated into the PHA polymer, which will impact the properties of the PHA produced. Based on the substrate specificities of PhaC and subunit composition, PhaCs are categorized into four main classes: class I, II, III and IV. Class I, III and IV PhaC prefer SCL-PHA monomer, whereas class II PhaC prefers MCL-PHA monomer. Class I and II PhaCs are single-unit enzymes. Class III consists of two subunits, PhaC and PhaE, while class IV consists of PhaC and PhaR (Rehm, 2003).

This present paper reviews the properties, metabolic pathways of P(3HB-*co*-3HHx) production, factors affecting the 3HHx molar composition in P(3HB-*co*-3HHx) copolymer, and potential applications of P(3HB-*co*-3HHx).

## Properties of P(3HB-*co*-3HHx)

**FIGURE 3:**
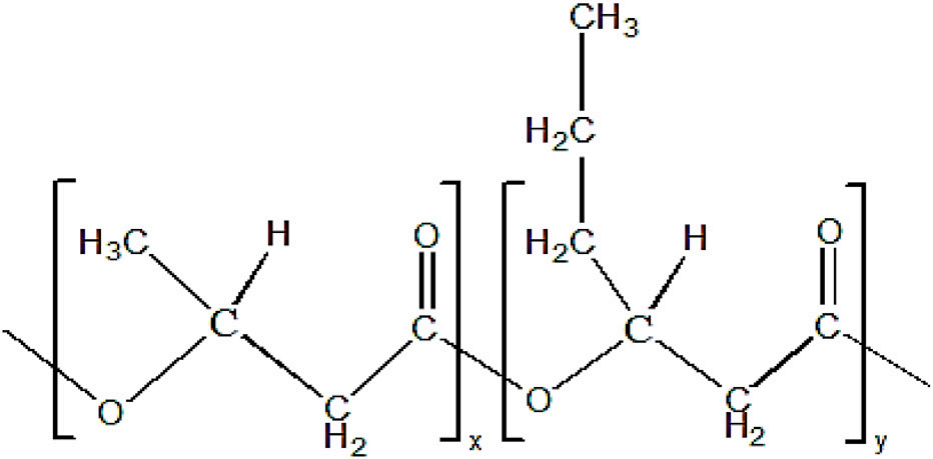
Chemical structure of P(3HB-*co*-3HHx). ‘x’ and ‘y’ indicate the repeating units of monomer.

P(3HB-*co*-3HHx) copolymer consists of two monomer compositions, which are 3HB and 3HHx. The chemical structure of P(3HB-*co*-3HHx) is shown in Figure 3.

P(3HB) is a homopolymer of SCL monomer unit consisting of four carbon atoms and is the most common type of PHA. It is reported that the properties of P(3HB) are approximately similar to commercial plastics such as polypropylene in terms of tensile strength and Young’s Modulus (Sudesh and Iwata, 2008). However, the elongation at break of P(3HB) is 5% which is way lower than polypropylene which is 400% (Sudesh et al., 2000). The melting temperature (*T*_m_) of P(3HB) is approximately 180°C, and the degree of crystallinity is 55–80% (Holmes, 1988). Due to its brittleness, stiffness and high *T*_m_, P(3HB) has poor processability and limits applications.

On the other hand, 3HHx is a MCL monomer unit that consists of six carbon atoms. Since 3HHx has a longer alkyl side chain, it cannot crystallize in the 3HB lattice and hence, avoiding the isodimorphism phenomenon. MCL-PHAs are amorphous in contrast to crystalline SCL-PHAs. MCL-PHAs exhibit elastomeric properties, soft, low *T*_m_, low glass transition temperature (*T*_g_), low tensile strength, and high elongation at break (Yu, 2007; Reddy et al., 2022).

The mixture of SCL- and MCL-PHA has superior thermal and mechanical properties than PHA homopolymers. Compared to SCL-PHA and MCL-PHA, the mixture of SCL- and MCL-PHA results in lower *T*_m_, lower degree of crystallinity, and higher elongation at break (Doi et al., 1995; Kellerhals et al., 2000; Noda et al., 2005).

The flexibility of P(3HB-*co*-3HHx) also depends on 3HHx monomer compositions (Shimamura et al., 1994). As 3HHx monomeric units increase, it becomes more flexible, softer, and has better processability. Hence, its improved properties and processability attract industrial attention for various applications, especially in packaging and single-use plastic.

The physicomechanical properties of P(3HB-*co*-3HHx) with different 3HHx molar fractions have been summarized in Table 1. In general, as the 3HHx molar fraction increases, the *M*_w_, *T*_m_, *T*_g_, Young’s modulus, and tensile strength decrease, whereas the elongation at break of the copolymer increases.

**TABLE 1:** The physicomechanical properties of P(3HB-*co*-3HHx).

3HHx composition (mol%)^a^	*T*_g_ (°C)^b^	*T*_m_ (°C)^c^	Tensile strength, MPa (Thickness)	Young’s modulus, MPa	Elongation at break, %	Molecular weight, Da			Source
						*M*_n_ (× 10^5^)^d^	*M*_w_ (× 10^5^)^e^	PDI^f^	
1 ± 0	ND	ND	ND	ND	ND	6.0 ± 0.0	15.3 ± 0.5	2.6 ± 0.1	Tan et al. (2020)∗
3 ± 0	ND	ND	ND	ND	ND	4.2 ± 0.2	14.0 ± 0.8	3.3 ± 0.3	Tan et al. (2020)∗
3 ± 0	ND	ND	ND	ND	ND	6.5 ± 0.2	17.3 ± 0.5	2.7 ± 0.0	Tan et al. (2020)∗
4	−1	164	ND	ND	ND	2.99 ± 0.04	5.47 ± 0.60	1.66	Murugan et al. (2017)∗
5	0	151	ND	ND	ND	1.0	1.9	1.9	Doi et al. (1995)
5	−3	125; 143	ND	ND	ND	4.6	15.8	3.3	Loo et al. (2005)
6 ± 0	ND	ND	ND	ND	ND	4.3 ± 0.2	12.7 ± 1.2	2.9 ± 0.1	Tan et al. (2020)∗
7	−1	132	ND	ND	ND	1.7	4.5	2.65	Foong et al. (2018)∗
7 ± 0	ND	ND	ND	ND	ND	5.9 ± 0.7	18.7 ± 1.7	3.2 ± 0.1	Tan et al. (2020)∗
10	−1	127	21 (0.1 mm)	ND	400	1.2	3.04	2.6	Doi et al. (1995)
11 ± 1	ND	ND	ND	ND	ND	2.5 ± 0.1	8.4 ± 1.2	3.3 ± 0.3	Tan et al. (2020)∗
11 ± 0	ND	ND	ND	ND	ND	5.9 ± 0.3	18.3 ± 1.9	3.1 ± 0.1	Tan et al. (2020)∗
12	ND	170	18.3 ± 1.8 (25–30 μm)	1286.4 ± 90.8	3.6 ± 0.1	0.14	0.44 ± 19	3.10 ± 0.05	Volova et al. (2016)
12	−9	163	ND	ND	ND	2.63 ± 0.16	6.01 ± 0.34	2.30	Murugan et al. (2017)∗
13	−9	160	ND	ND	ND	ND	ND	ND	Murugan et al. (2016)∗
13 ± 0	ND	ND	ND	ND	ND	2.2 ± 0.2	8.0 ± 0.2	3.6 ± 0.2	Tan et al. (2020)∗
14 ± 1	ND	ND	ND	ND	ND	3.3 ± 0.1	7.7 ± 0.4	2.3 ± 0.0	Tan et al. (2020)∗
15	0	115	23 (0.1 mm)	ND	760	2.1	7.92	3.7	Doi et al. (1995)
15	−12	156	ND	ND	ND	0.39 ± 0.04	6.85 ± 0.65	1.77	Murugan et al. (2017)∗
16	0.9	116.1; 131.5	ND	ND	ND	2.89 ± 0.16	4.45 ± 0.46	1.5 ± 0.1	Thinagaran and Sudesh (2019)∗
17	−2	130	ND	ND	ND	6.62	11.92	1.8	Shimamura et al. (1994)
17	−2	120	20 (0.1 mm)	ND	850	5.1	11.22	2.2	Doi et al. (1995)
17	ND	ND	ND	ND	ND	1.05 ± 0.40	2.60 ± 0.52	2.50	Murugan et al. (2017)∗
17	−1.6	113.4; 129.5	ND	ND	ND	2.76 ± 0.08	4.37 ± 0.54	1.6 ± 0.1	Thinagaran and Sudesh (2019)∗
18	−22	167	ND	ND	ND	2.6	8.6	3.3	Foong et al. (2018)∗
18	−0.3	110.6; 125.6	ND	ND	ND	2.40 ± 0.07	3.58 ± 0.09	1.5 ± 0.0	Thinagaran and Sudesh (2019)∗
18	ND	ND	ND	ND	ND	3.2 ± 0.2	6.9 ± 0.7	2.2 ± 0.1	Tan et al. (2020)∗
19	−4	111	ND	ND	ND	0.4	3.32	8.3	Doi et al. (1995)
19	0	145	ND	ND	ND	ND	ND	ND	Murugan et al. (2016)∗
19	ND	ND	ND	ND	ND	1.57 ± 0.00	3.30 ± 0.00	2.10	Murugan et al. (2017)∗
19	−1.7	99.6; 113.3	ND	ND	ND	1.84 ± 0.19	2.77 ± 0.31	1.5 ± 0.0	Thinagaran and Sudesh (2019)∗
20	−4.79	107.72	ND	ND	ND	4.4	7.5	1.7	Purama et al. (2018)∗
24	−2.0	109.7	ND	ND	ND	1.81 ± 0.30	2.7 ± 0.42	1.5 ± 0.1	Thinagaran and Sudesh (2019)∗
24.6	ND	167	21.6 ± 0.8 (25–30 μm)	1207.5 ± 21.4	4.1 ± 0.1	0.11	0.60	5.42	Volova et al. (2016)
25	−4	52	ND	ND	ND	2.12	7.42	3.5	Doi et al. (1995)
27	−1	120	ND	ND	ND	ND	ND	ND	Murugan et al. (2016)∗
28	−2.29	85.33	ND	ND	ND	3.2	5.8	1.8	Purama et al. (2018)∗
32	−1	88	8 ± 1	101 ± 6	856 ± 21	2.24 ± 0.20	3.47 ± 0.18	1.55 ± 0.06	Wong et al. (2012)∗
43	−4	86	5 ± 1	75 ± 9	481 ± 47	0.72 ± 0.05	1.17 ± 0.06	1.63 ± 0.03	Wong et al. (2012)∗
43	ND	176	19.0 ± 0.3 (25–30 μm)	938.0 ± 14.1	5.0 ± 0.1	0.08	0.31	3.88	Volova et al. (2016)
55	ND	171	6.6 ± 0.3 (25– 30 μm)	311.4 ± 21.8	13.6 ± 0.3	0.17	0.70	4.10	Volova et al. (2016)
56	−6	86	1 ± 1	12 ± 2	368 ± 1	0.82 ± 0.06	1.20 ± 0.10	1.45 ± 0.01	Wong et al. (2012)∗
60	−11	ND	<1 ± 1	3 ± 1	424 ± 23	1.26 ± 0.03	2.11 ± 0.13	1.75 ± 0.07	Wong et al. (2012)∗
65.7	ND	173	7.8 ± 0.7 (25–30 μm)	209.5 ± 17.0	140.6 ± 5.5	0.17	0.68	3.90	Volova et al. (2016)
68	ND	172	7.4 ± 0.7 (25–30 μm)	217.0 ± 11.3	177.0 ± 4.8	0.14	0.72	4.84	Volova et al. (2016)
70	−12	ND	<1 ± 1	<1 ± 0	1075 ± 158	1.37 ± 0.16	2.27 ± 0.2	1.66 ± 0.03	Wong et al. (2012)∗

^a^ 3HHx, 3-hydroxyhexanoate.

^b^ *T*_g_, glass transition temperature.

^c^ *T*_m_, melting temperature.

^d^ *M*_n_, number average molecular weight.

^e^ *M*_w_, weight average molecular weight.

^f^ PDI, polydispersity index (*M*_w_/*M*_n_); ND, not detected. Asterisk (∗) indicates the data are obtained from Ecobiomaterial Laboratory, School of Biological Sciences, Universiti Sains Malaysia, 11800, Pulau Pinang, Malaysia.

## Biodegradability of P(3HB-*co*-3HHx)

Previous research has established that P(3HB-*co*-3HHx) can be biodegraded in aerobic environments such as seawater, freshwater, soil, and domestic compost (Morse et al., 2011). Under aerobic conditions, P(3HB-*co*-3HHx) is degraded into water and carbon dioxide. On the other hand, anaerobic biodegradation has also been carried out using PHB depolymerase, whereby P(3HB-*co*-3HHx) is degraded into water and methane (Abe and Doi, 1999; Lee and Choi, 1999). P(3HB-*co*-3HHx) takes around six months to be degraded in seawater (KANEKA Corporation, 2019). There are a few factors that affect the biodegradation of PHA copolymers, such as the environment, the microbial community, types of PHA monomer composition, temperature, and humidity.

The rate of PHA degradation increases as the degree of crystallinity decreases (Morse et al., 2011). Morse and co-workers reported that the films made from P(3HB-*co*-3HHx) with 10 mol% of 3HHx had a faster degradation rate than those with 3.8 mol% of 3HHx. Furthermore, Volova and co-workers reported that the biodegradability of P(3HB-*co*-3HHx), a PHA copolymer was faster than P(3HB), a PHA homopolymer (Volova et al., 2017). This is because P(3HB) has higher crystallinity than P(3HB-*co*-3HHx), leading to a lower degradation rate. Moreover, it was reported that the rate of enzymatic degradation of P(3HB-*co*-3HHx) film by P(3HB) depolymerase gets higher as the 3HHx content increases to 15 mol% (Shimamura et al., 1994). When the 3HHx mol% in the P(3HB-*co*-3HHx) increases, the degree of crystallinity of P(3HB-*co*-3HHx) decreases, thereby increasing the biodegradation rate of the polymer. It is also reported that the degradation rate of the amorphous polymer was faster than that of highly crystalline polymer (Molitoris et al., 1996).

Besides that, Wang and co-workers reported that P(3HB-*co*-3HHx) polymer surface morphology could affect its biodegradation rate. Molitoris and co-workers reported that the degradation was faster on PHA with fissures on its surface than on PHA with a smooth surface (Molitoris et al., 1996). A porous and rough surface tends to have a faster degradation rate as it facilitates the attachment of lipase or bacteria to the film to begin the degradation process (Wang et al., 2004).

## Metabolic pathway of P(3HB-*co*-3HHx) production

**FIGURE 4:**
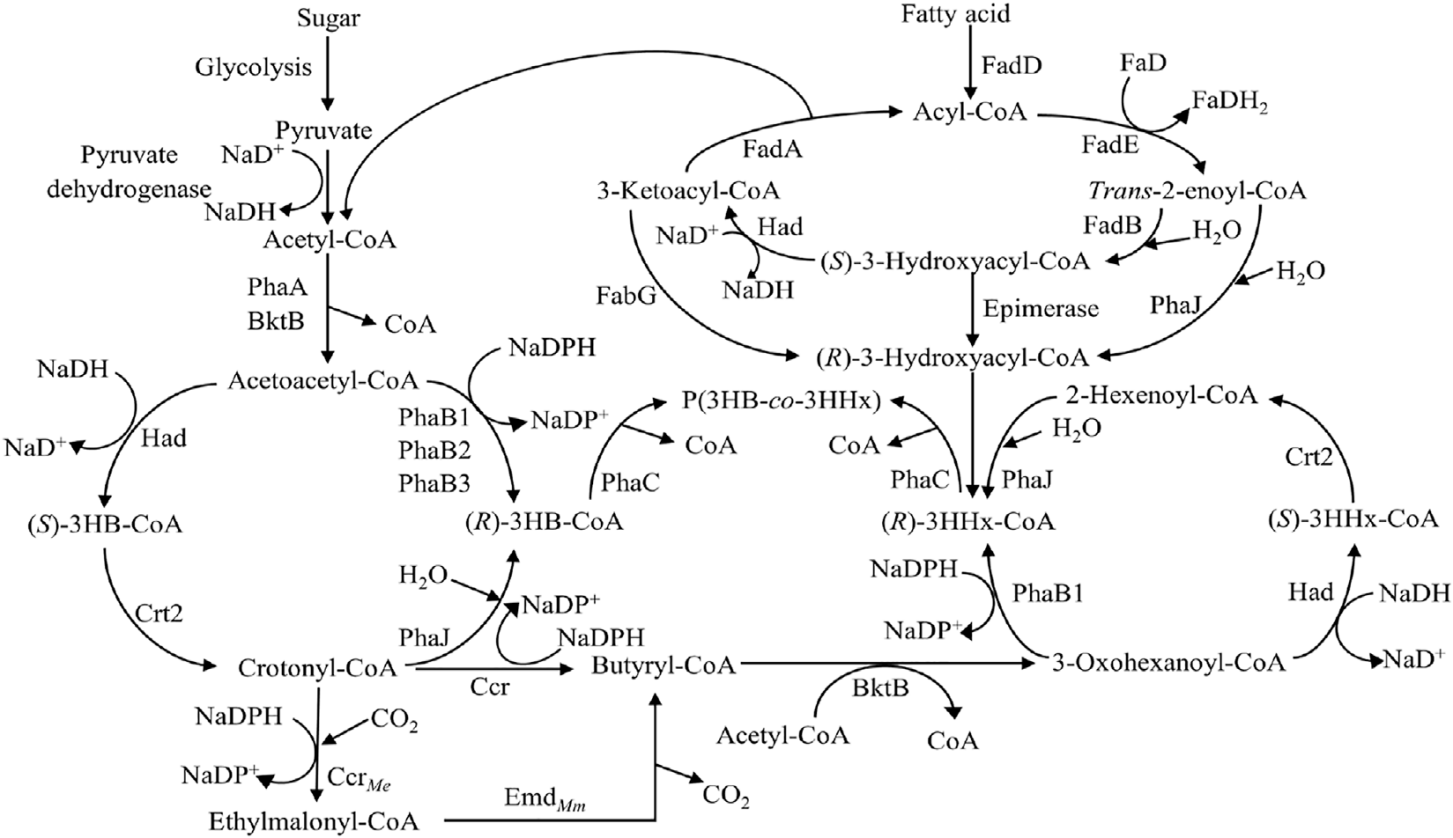
Metabolic pathway of P(3HB) and P(3HB-*co*-3HHx) production (Sudesh et al., 2000; Tsuge, 2002; Zhang et al., 2019). Abbreviation: PHA, polyhydroxyalkanoate; PhaA, *β*-ketothiolase; PhaB, NADPH-dependent acetoacetyl-CoA reductase; PhaC, PHA synthase; FadA, 3-ketoacyl-CoA thiolase; FadB, enoyl-CoA hydratase; FadD, acyl-CoA synthetase; FadE, acyl-CoA dehydrogenase; FabG, 3-ketoacyl-CoA reductase; 3HB-CoA, 3-hydroxybutyryl-CoA; 3HHx-CoA, 3-hydroxyhexanoyl-CoA; PhaJ, (*R*)-specific enoyl-CoA hydratase; Had, 3-hydroxyacyl-CoA dehydrogenase; Crt2, crotonase; Ccr, crotonyl-CoA reductase; Ccr_*Me*_, crotonyl-CoA carboxylase from *Methylorubrum extorquens*; Emd_*Mm*_, ethylmalonyl-CoA decaroboxylase from *Mus musculus*.

The most common PHA biosynthesis pathway consists of three genes which are *β*-ketothiolase (PhaA) coded by *phaA* gene, NADPH-dependent acetoacetyl-CoA reductase (PhaB) coded by *phaB* gene and PhaC coded by *phaC* gene (Anderson and Dawes, 1990). The acetyl-CoA from various pathways like fatty acid β-oxidation, glycolysis, and many more will first be converted to acetoacetyl-CoA by PhaA. The acetoacetyl-CoA will then be reduced to (*R*)-3-hydroxybutyryl-CoA [(*R*)-3HB-CoA] by PhaB and followed by incorporation into PHA by PhaC (Figure 4).

When fatty acids are used as carbon sources, fatty acids will be converted to acyl-CoA by acyl-CoA synthetase (FadD). Acyl-CoA will be oxidized to *trans*-enoyl-CoA catalyzed by acyl-CoA dehydrogenase (FadE) followed by hydration into (*S*)-3-hydroxyacyl-CoA by enoyl-CoA hydratase (FadB). (*S*)-3-hydroxyacyl-CoA will then be oxidized into 3-ketoacyl-CoA by 3-hydroxyacyl-CoA dehydrogenase (Had). Lastly, 3-ketoacyl-CoA thiolase (FadA) will convert 3-ketoacyl-CoA into acyl-CoA with two carbon atoms lesser, releasing one acetyl-CoA. This acetyl-CoA can be converted to PHA using the previous pathway. Six carbons of (*S*)-3-hydroxyacyl-CoA or (*S*)-3-hydroxyhexanoyl-CoA [(*S*)-3HHx-CoA] will be converted into (*R*)-3-hydroxyhexanoyl-CoA [(*R*)-3HHx-CoA] by (*R*)-specific enoyl-CoA hydratase (PhaJ). (*R*)-3HHx-CoA will then be incorporated into P(3HB-*co-*3HHx) by PhaC (Figure 4) (Tsuge, 2002).

Another engineered pathway involving the formation of P(3HB-*co*-3HHx) is the fatty acids *de novo* synthesis pathway, where sugar is used as the sole carbon source. This pathway begins with sugar which will be converted to pyruvate via glycolysis and decarboxylated into acetyl-CoA by pyruvate dehydrogenase. The acetyl-CoA will be converted into acetoacetyl-CoA by PhaA. The acetoacetyl-CoA formed will be reduced to (*S*)-3-hydroxybutyryl-CoA [(*S*)-3HB-CoA] by Had followed by conversion to crotonyl-CoA by crotonase (Crt2). Crotonyl-CoA will either be converted to (*R*)-3HB-CoA by PhaJ or butyryl-CoA. The (*R*)-3HB-CoA will be incorporated into P(3HB-*co-*3HHx) as 3HB. The crotonyl-CoA will be reduced to butyryl-CoA by crotonyl-CoA reductase (Ccr). The butyryl-CoA will be converted to 3-oxohexanoyl-CoA followed by reduction to (*S*)-3HHx-CoA by Had. (*S*)-3HHx-CoA will be converted to 2-hexenoyl-CoA by Crt2 and then hydrated to (*R*)-3HHx-CoA by PhaJ. (*R*)-3HHx-CoA will be incorporated into P(3HB-*co*-3HHx) by PhaC as 3HHx (Figure 4) (Zhang et al., 2019).

## Factors affecting the 3-hydroxyhexanoate (3HHx) compositions of P(3HB-*co*-3HHx)

### PhaCs

One of the main factors affecting the 3HHx molar composition is the PhaC. PhaC is the most important protein in PHA biosynthesis because it determines the type of PHA produced (Sudesh et al., 2000). Hence, many researchers have dedicated their time in search of a good naturally occurring PhaC.

As mentioned above in the introduction, class I, III, and IV PhaCs could only incorporate SCL-PHA monomers, while class II PhaCs could incorporate MCL-PHA. There is also a particular group of class I PhaC that will incorporate both SCL- and MCL-PHA monomers into the PHA polymer produced (Neoh et al., 2022). For example, PhaCs from *A. caviae* (PhaC_*Ac*_), *Rhodococcus aetherivorans* I24 (PhaC1_*Ra*_ and PhaC2_*Ra*_), PhaC from mangrove soil (PhaC_BP-M-CPF4_), *Chromobacterium* sp. USM2 (PhaC_*C*s_) and *Pseudomonas* sp. 61–3 (PhaC1_*Ps*_ and PhaC2_*Ps*_) (Doi et al., 1995; Fukui and Doi, 1997; Matsusaki et al., 1998; Bhubalan et al., 2010; Budde et al., 2011; Foong et al*.*, 2018).

*A. caviae* was one of the first bacterial strains to be discovered to produce P(3HB-*co*-3HHx) (Shimamura et al., 1994). PhaC_*Ac*_ possesses substrate specificity for copolymerizing both 3HB-CoA and 3HHx-CoA into P(3HB-*co*-3HHx) copolymer using alkanoic acids and olive oil (Doi et al., 1995). Heterologous expression of PhaC_*Ac*_ in *C. necator* mutant, PHB^−^4, and *Pseudomonas putida* GPp104 could produce P(3HB-*co*-3HHx) with a maximum 22 mol% of 3HHx using octanoate and 40 mol% of 3HHx using hexanoate respectively (Fukui & Doi, 1997). The recombinant strain *A. eutrophus* (PHB^−^4/pJRDEE32d13) harboring *phaC*_*Ac*_ could produce P(3HB-*co*-3HHx) with 4 mol% of 3HHx content using olive oil (Fukui and Doi, 1998). Kahar and co-workers reported that recombinant strain *C. necator* PHB^−^4 harboring pJRDEE32d13 inserted with *phaC*_*Ac*_ could accumulate P(3HB-*co*-3HHx) with 5 mol% of 3HHx content from soybean oil (Kahar et al., 2004).

Budde and co-workers reported two different PhaCs, PhaC1_*Ra*_ and PhaC2_*Ra*_, in *R. aetherivorans* I24 (Budde et al., 2011). The heterologous expression of PhaC1_*Ra*_ and PhaC2_*Ra*_ in recombinant *C. necator* could produce P(3HB-*co*-3HHx). The study showed that recombinant *C. necator* strains, Re2000 and Re 2001, expressing PhaC1_*Ra*_ and PhaC2_*Ra*_, respectively, could produce P(3HB-*co*-3HHx) using hexanoate and octanoate. Re2000 and Re2001 could produce 11.5 mol% of 3HHx and 18.9 mol% of 3HHx from hexanoate, respectively, while producing 6.6 mol% of 3HHx and 10.4 mol% of 3HHx from octanoate, respectively (Budde et al., 2011). This result showed that PhaC2_*Ra*_ produced P(3HB-*co*-3HHx) with higher 3HHx content than PhaC1_*Ra*_ from fatty acids.

PhaC_BP-M-CPF4_ was discovered in mangrove soil metagenome at Balik Pulau, Malaysia. It has wide substrate specificity as it can incorporate both SCL-PHA and MCL-PHA. The heterologous expression of PhaC_BP-M-CPF4_ in *C. necator* PHB^−^4 produced P(3HB-*co*-3HHx) with 7 mol% of 3HHx from CPKO and 18 mol% of 3HHx when co-fed with fructose and sodium hexanoate (Foong et al., 2018). Besides *C. necator* PHB^−^4, another *C. necator* PHA-negative mutant strains, H16ΔC, Re2058, and Re2160 harboring *phaC*_BP-M-CPF4_ were reported to be able to produce P(3HB-*co*-3HHx) ranging from 3–18 mol% of 3HHx from CPKO (Tan et al., 2020).

*Chromobacterium* sp. strain USM2 was discovered in Langkawi, Malaysia (Yong, 2009). It was reported that PhaC_*C*s_ heterologous expressed in *C. necator* PHB^−^4 could synthesize P(3HB-*co*-3HHx) copolymer with 4 mol% of 3HHx using CPKO (Bhubalan et al., 2010). In addition, *Pseudomonas* sp. 61–3 isolated from soil possesses two different PhaCs, PhaC1_*Ps*_ and PhaC2_*Ps*_. It could incorporate 3HB and 3HA units of C_4_ and C_12_ when supplemented with sugars and alkanoic acids. The heterologous expression of PhaC1_*Ps*_ and PhaC2_*Ps*_ in *P. putida* GPp104 and *C. necator* PHB^−^4 could produce P(3HB-*co*-3HHx) with 3HHx ranging from 1 to 16 mol% from alkanoic acids (Matsusaki et al., 1998).

Based on the above, it is clear that PhaCs can affect the 3HHx molar composition of P(3HB-*co*-3HHx) produced. The difference in 3HHx molar composition is due to the substrate specificity of the PhaC towards 3HHx. Some PhaCs with higher substrate specificity towards 3HHx tend to produce P(3HB-*co*-3HHx) with higher 3HHx, and some PhaCs with lower substrate specificity towards 3HHx will lead to lower 3HHx. This may be due to the substrate entrance channel and the binding pocket of the PhaC where folding of the amino acid forming both substrate entrance channel and binding pocket are better in binding to 3HHx-CoA and hence, incorporating into P(3HB-*co*-3HHx). However, this is yet to be reported as to date, there is no co-crystal structure of (*R*)-3HHx-CoA with PhaC, but there is a co-crystal structure of PhaCs with CoA, which was reported by Chek and co-workers elucidating how the CoA group is bound to the binding pocket of PhaC_*C*s_-CAT (Chek et al., 2020). The comparison of the production titers of the P(3HB-*co*-3HHx) from different researchers is shown in Table 2.

**Table 2:** Production titers of P(3HB-*co*-3HHx).

Bacterial strains	Carbon sources	PHA content (wt%)	3HHx monomer composition (mol%)	Production scale	References
*A. caviae*	Olive oil	ND	17	3 L fermenter	Shimamura et al. (1994)
*A. caviae*	Alkanoic acids and olive oil	27	25	500 ml flask	Doi et al. (1995)
*Pseudomonas putida* GPp104 harboring *phaC*_*Ac*_	Hexanoate	38	40	500 ml flask	Fukui and Doi (1997)
*A. eutrophus* (PHB^−^4/pJRDEE32d13) harboring *phaC*_*Ac*_	Olive oil	76	4	500 ml flask	Fukui and Doi (1998)
*P. putida* GPp104 harboring *phaC1*_*Ps*_	Alkanoic acids	43	16	500 ml flask	Matsusaki et al. (1998)
*C. necator* PHB^−^4 harboring *phaC-J*_*Ac*_	Fructose	39	1.6	500 ml flask	Fukui et al. (2002)
*C. necator* PHB^−^4 harboring pJRDEE32d13 harboring *phaC*_*Ac*_	Soybean oil	71–74	5	10 L fermenter	Kahar et al. (2004)
*C. necator* strains, Re2001 harboring *phaC2*_*Ra*_	Hexanoate	48	18.9	250 ml flask	Budde et al. (2011)
*C. necator* Re2058/pCB113	Palm oil	73	19	2 L fermenter	Riedel et al*.* (2012)
*C. necator* Re2160/pCB113	CPKO	45	68	250 ml flask	Wong et al. (2012)
*C. necator* Re2058/pCB113	PO	67	27	13 L fermenter	Murugan et al. (2017)
*C. necator* PHB^−^4 harboring *phaC*_BP-M-CPF4_	Sodium hexanoate	44	18	250 ml flask	Foong et al. (2018)
*C. necator* Re2058/pCB113	Date seed oil and date molasses	10	28	250 ml flask	Purama et al. (2018)
*C. necator* Re2058/pCB113	SPO	33	34	250 ml flask	Thinagaran and Sudesh (2019)
*C. necator* Re2160/pHT1-*C*_BP-M-CPF4_	CPKO	63.3	18	250 ml flask	Tan et al. (2020)
*C. necator* PHB^−^4 harboring *phaC*_*C*s_	CPKO	63	4	250 ml flask	Bhubalan et al. (2010)

CPKO, crude palm kernel oil; PO, palm olein; SPO, sludge palm oil; ND, not detected.

### Engineering of PhaCs

Besides naturally occurring PhaCs, engineering existing PhaCs could also affect the 3HHx molar composition in P(3HB-*co*-3HHx). *In vitro* evolution system can be used to improve the activity and broaden the substrate specificity of PhaC.

Kichise and co-workers had selected PhaC_*Ac*_ for *in vitro* evolution as it can produce P(3HB-*co*-3HHx) copolymer from plant oils or alkanoic acid. *In vitro* evolution of PhaC_*Ac*_ was carried out using error-prone PCR, and as a result, two PhaC_*Ac*_ mutants (E2-50 and T3-11) were obtained, which had improved 3HHx incorporation. The PhaC_*Ac*_ mutants (E2-50 and T3-11) in *E. coli* LS5218 could synthesize P(3HB-*co*-3HHx) with higher 3HHx content, which was 18 mol% of 3HHx and 16 mol% of 3HHx, respectively compared to wild-type with 10 mol% of 3HHx using sodium dodecanoate. P(3HB-*co*-3HHx) accumulated was 3-fold higher than the wild-type PhaC_*Ac*_. It was discovered that E2-50 and T3-11 have the single amino acid substitution of N149S and D171G, respectively (Kichise et al., 2002).

Tsuge and co-workers further studied the synergistic effects of PhaC_*Ac*_ mutants, N149S and D171G double mutation (NSDG) in *C. necator* PHB^−^4 for PHA production. The NSDG mutant was able to produce P(3HB-*co*-3HHx) with 3HHx as high as 18.1 mol%, which was higher than the wild type with 12.2 mol% using sodium octanoate. Besides sodium octanoate, the NSDG mutant could also produce P(3HB-*co*-3HHx) with 5.2 mol% of 3HHx from soybean oil, which was higher than the wild type with only 3.5 mol% of 3HHx. The NSDG mutant could also produce P(3HB-*co*-3HHx) with 2.90 × 10^6^ Da from sodium octanoate, which is near to ultrahigh molecular weight PHA (UHMW-PHA), and it is suitable for making strong films and fibers (Iwata, 2005; Tsuge et al., 2007). N149S mutation increased the incorporation of 3HA into PHA polymer and the molecular weight of the polymer, whereas D171G mutation increased PHA accumulation. Hence, the synergistic effect of N149S and D171G double mutation on PHA production was higher than predicted (Tsuge et al., 2007).

PhaC_*Cn*_ belongs to the type I PHA synthase, which shows substrate specificity towards (*R*)-3HA-CoAs with the acyl chain length of C3–C5. The alanine residue at position 510 may define the substrate specificity and the properties of the enzyme. Tsuge and co-workers found out that the performance of saturation point mutagenesis at position 510 in PhaC_*Cn*_ using a PCR-based method could drastically impact the substrate specificity and polymerization activity for *in vivo* PHA biosynthesis in *E. coli* and *C. necator* PHB^−^4. The co-expression of the mutant PhaC_*Cn*_ and PhaJ_*Ac*_ in *E. coli* LS5218 in the presence of fatty acids could produce P(3HB-*co*-3HHx) with 0.7 mol% 3HHx content, which was higher than that of the wild-type with only 0.2 mol%. Besides *E. coli*, *C. necator* PHB^−^4 harboring mutant PhaC_*Cn*_ produced P(3HB-*co*-3HHx) with as high as 1.6 mol% 3HHx compared to the wild type of 1.2 mol%. This study also showed that the molecular weight of PHA produced by mutant PhaC_*Cn*_ was higher than the wild type (Tsuge et al., 2004). In addition, Chuah and co-workers reported that saturation point mutagenesis at position A479 in PhaC_*C*s_ could enhance substrate specificity for 3HHx. A479 in PhaC_*C*s_ corresponded to A510 of PhaC_*Cn*_ and was selected as the site of saturation point mutagenesis. It showed that the A479S mutation could increase 3HHx content to 6.6 mol%, 4-fold higher than the wild-type with only 1.6 mol% 3HHx. Besides that, mutation of A479T and A479G showed a 2.8-fold and 1.6-fold increment of 3HHx compared to wild-type PhaC_*C*s_ (Chuah et al., 2013).

### Metabolic engineering of PHA-producing strains

Another factor affecting the 3HHx molar composition is metabolic engineering through the genetic modification of microbial strains. It can involve genes from various pathways, including PHA-related and non-PHA-related genes.

Firstly, the deletion of *phaB* in the *pha* operon could affect the 3HHx monomer supply and hence, affect the 3HHx molar composition of the P(3HB-*co*-3HHx) produced. Budde and co-workers reported that the deletion of *phaB* from the genome of *C. necator* could result in the disruption of the 3HB-CoA synthesis pathway (Budde, 2010). Based on Figure 4, PhaB is an important enzyme for supplying (*R*)-3HB-CoA from acetoacetyl-CoA due to its high catalytic efficiency and expression (Zhang et al., 2019). Budde and co-workers reported that *C. necator* strains Re2058 and Δ*phaB* genes mutant, Re2160, were evaluated for their performances through high cell density P(3HB-*co*-3HHx) production from palm oil. As a result, Re2058 and Re2160 accumulated 17 mol% of 3HHx and 30 mol% of 3HHx, respectively (Budde et al., 2011). Disruption in the 3HB-CoA synthesis pathway leads to lower 3HB-CoA and hence increases the 3HHx molar compositions in the P(3HB-*co*-3HHx) copolymer produced.

Besides that, modification of the acetoacetyl-CoA reduction step in engineered *C. necator* could affect the 3HHx molar composition in P(3HB-*co*-3HHx) from sugars. Zhang and co-workers managed to produce P(3HB-*co*-3HHx) with 22 mol% of 3HHx content from glucose using *C. necator* strain NSDG-GG harboring *phaJ4a* with Δ*phaB1* because deletion of *phaB1* affected the flux for (*R*)-3HB-CoA and (*R*)-3HHx-CoA formation. They introduced *had* and *crt2* into the *phaB* locus in the *pha* operon to increase the 3HHx monomer composition without significantly affecting PHA synthesis. Based on Figure 4, acetyl-CoA can be converted to 2-hexenoyl-CoA via fatty acid *de novo* synthesis pathway. In that pathway, acetoacetyl-CoA will be converted to crotonyl-CoA by Had and Crt2. At this stage, crotonyl-CoA will be hydrated to (*R*)-3HB-CoA by PhaJ, or crotonyl-CoA will be reduced to butyryl-CoA by Ccr, respectively. Butyryl-CoA will then be converted to 2-hexenoyl-CoA to produce (*R*)-3HHx-CoA. Zhang and co-workers have further enhanced the fatty acid *de novo* synthesis pathway by adding crotonyl-CoA carboxylase from *Methylorubrum extorquens* (Ccr_*Me*_) and ethylmalonyl-CoA decarboxylase from *Mus musculus* (Emd_*Mm*_) (Zhang et al., 2019). Both Ccr_*Me*_ and Emd_*Mm*_ create an alternative pathway towards the formation of butyryl-CoA, which will eventually be converted to (*R*)-3HHx-CoA and hence, increasing the 3HHx molar composition in the P(3HB-*co*-3HHx) copolymer produced (Figure 4).

In addition, the deletion of FadB will disrupt (*R*)-3HB-CoA synthesis, leading to the formation of (*R*)-3HHx-CoA by PhaJ. Insomphun and co-workers reported that the disruption of *fadB1* in engineered *C. necator* harboring *phaJ* could enhance the (*R*)-3HHx-CoA supply from 6-carbons *trans*-2-enoyl-CoA (2-hexenoyl-CoA) catalyzed by PhaJ and increase the copolymerization of (*R*)-3HHx-CoA in P(3HB-*co*-3HHx) copolymer. It was shown that 3HHx monomer composition was increased by around 1–1.5 mol% (Insomphun et al., 2014). Originally, *trans*-2-enoyl-CoA formed will be converted to (*S*)-3-hydroxyacyl-CoA or (*R*)-3-hydroxyacyl-CoA by FadB and PhaJ, respectively. When *trans*-2-enoyl-CoA is catalyzed by FadB, it is converted to (*S*)-3-hydroxyacyl-CoA, which will lead to the formation of acyl-CoA and acetyl-CoA, and eventually, the acetyl-CoA formed will be converted to (*R*)-3HB-CoA. (*R*)-3HB-CoA will then be incorporated into P(3HB-*co*-3HHx) as 3HB, hence might lower the 3HHx molar composition in P(3HB-*co*-3HHx). When FadB is deleted, there will be no formation of (*S*)-3-hydroxyacyl-CoA, hence favoring the hydration of 2-hexenoyl-CoA to form (*R*)-3HHx-CoA (Figure 4). This will increase the ratio of (*R*)-3HHx-CoA to (*R*)-3HB-CoA, leading to higher 3HHx molar composition.

Besides that, the fatty acid β-oxidation pathway could be modified to increase 3HHx molar composition by inserting 3-ketoacyl-CoA reductase of *E. coli* (FabG_*Ec*_). Taguchi and co-workers co-expressed *phaC*_*Ac*_ and *fabG*_*Ec*_ in *E. coli,* and as a result, the 3HHx molar composition of P(3HB-*co*-3HHx) increased from 0 to 14 mol%. Besides that, they also co-expressed a class II PhaC, PhaC from *Pseudomonas* sp. 61–3, which is known to have substrate specificity towards MCL-PHA monomer with *fabG*_*Ec*_, resulting in an increase of 3HHx from 0 to 12 mol% (Figure 4) (Taguchi et al., 1999). This is probably due to the ability of FabG_*Ec*_ to catalyze the conversion of 6-carbons of 3-ketoacyl-CoA to (*R*)-3HHx-CoA which will then be incorporated into P(3HB-*co-*3HHx) by PhaC.

Furthermore, the overexpression of PhaJ using plasmid could enhance 3HHx monomer composition in P(3HB-*co*-3HHx). Kawashima and co-workers reported that insertion of *phaJ*_*Ac*_ with *phaC*_NSDG_ in *C. necator* strain could produce 10.5 mol% of 3HHx, which is 6.6-fold higher than the *C. necator* strain harboring *phaC*_NSDG_ only (Kawashima et al., 2015). Tan and co-workers also co-expressed *phaJ1* from *Pseudomonas aeruginosa* with *phaC*_BP-M-CPF4_ in *C. necator* transformants which could produce 3HHx as high as 14 mol%, which is higher than *C. necator* transformants harboring *phaC*_BP-M-CPF4_ with only 6 mol% (Tan et al., 2020). PhaJ is involved in fatty acid β-oxidation, whereby it creates an alternative pathway for supplying (*R*)-3HHx-CoAs to be incorporated into P(3HB-*co*-3HHx), hence increasing the 3HHx molar composition. It catalyzes the hydration of (*R*)-2-hexenoyl-CoA from fatty acid β-oxidation and hydrates them to (*R*)-3HHx-CoA (Figure 4) (Fukui et al., 1998). This showed that the expression of PhaJ could lead to more (*R*)-3HHx-CoA production by enhancing the channeling pathway from fatty acid β-oxidation and hence, increasing 3HHx mol% in P(3HB-*co*-3HHx). Besides that, expression of PhaJ from *Streptomyces* sp. CFMR7 (PhaJ_*S*s_) was reported to increase the 3HHx molar composition from 4 mol% and 7 mol% to 12 mol% and 18 mol% using palm oil and crude palm oil, respectively (Tan et al., 2022).

## Applications of P(3HB-*co*-3HHx)

P(3HB-*co*-3HHx) has been extensively utilized in making straws, shopping bags, cutlery, containers, food packaging, coffee capsules, fishery items, biomedicine and adhesive due to its biodegradability and lack of toxicity (Qiu et al., 2021). Table 3 shows P(3HB-*co*-3HHx) production strains, carbon sources, the name of the company, the production scale, and their applications.

**TABLE 3:** Commercialized P(3HB-*co*-3HHx), its production strains, carbon sources, the name of company, production scale, and applications.

Production strains	Carbon sources	Company	Scale (ton/year)	Applications	References
*A. caviae*, recombinant *C. necator*	Natural oils from canola and soy	Danimer Scientific, United States	10,000	Bottles, recyclable paper, and board products	Danimer Scientific, (2022)
Recombinant *C. necator*	Vegetable oils	KANEKA, Japan	5000	Straws, cutlery, shopping bags, makeup container, and packaging materials	KANEKA The Dreamology Company, (2022)
Recombinant *C. necator*	Waste cooking oil	RWDC, Singapore and United States	4000	Cutlery, cups, bags, utensils, food containers, and drinking straws	RWDC Industries, (2022)
Recombinant *C. necator*	Crops and kitchen waste	Bluepha, China	1000	Cutlery, straw, packaging, coating, textiles, polymer films, 3D printing inks, and aquarium water restoration	Bluepha, (2022)

KANEKA Corporation has collaborated with Seven-Eleven Japan Corporation and THE NORTH FACE cafes to use straws made of P(3HB-*co*-3HHx) in the cafe area. Cutlery made from P(3HB-*co*-3HHx) by KANEKA Corporation is also available at FamilyMart convenience stores to promote the utilization of environmental-friendly materials (KANEKA Corporation, 2021a). Moreover, ITO EN Corporation has used the telescopic straws of “Oi Ocha Tea,” made of P(3HB-*co*-3HHx) and sold in Japan’s supermarkets (KANEKA Corporation, 2021b). Furthermore, JALUX corporation adopted shopping bags made of P(3HB-*co*-3HHx) in the BLUE SKY stores at Japan’s airports (KANEKA Corporation, 2021c).

Besides that, KANEKA has collaborated with Shiseido Company to use P(3HB-*co*-3HHx) copolymer as cosmetics packaging material. For example, the case of AquaGel Lip Palette is made of biodegradable P(3HB-*co*-3HHx), and this product has been sold since the 2020s (KANEKA Corporation, 2020). P(3HB-*co*-3HHx) is also applied in compostable capsules. Capsul’ in Pro, a manufacturer, has launched a Zero Impact capsule where the capsule is made of 100% biobased P(3HB-*co*-3HHx) derived from plant oils (Magazine, 2021). It provides an oxygen barrier to protect the flavor and aroma of coffee for 12 months. However, this capsule is still under development and has not been published.

Due to its biocompatibility and bioresorbable, the P(3HB-*co*-3HHx) copolymer is also suitable to be used as a scaffold for tissue engineering (Chang et al., 2014). It can be biocompatible with various cell types: smooth muscle cells, fibroblasts, osteoblasts and bone marrow cells (Qu et al., 2006a; Yang et al., 2011; Yu et al*.*, 2012). To test *in vitro* biocompatibilities, rabbit bone marrow cells were injected into P(3HB-*co*-3HHx) 3D scaffolds, and it was discovered that P(3HB-*co*-3HHx) performed best in terms of bone marrow cell attachment and proliferation (Chen and Wu, 2005). Scaffolds provide physical support and an artificial extracellular matrix (ECM) that promotes cell adhesion, proliferation and differentiation (Bryant and Anseth, 2003). Zhao and co-workers reported that P(3HB-*co*-3HHx) composite scaffolds produced *via* the 3D printing technique could improve their bioactive and osteogenic characteristics by enhancing the regeneration of bone in the defected calvarium of rats (Zhao et al., 2014). Ang and co-workers reported that blending of P(3HB-*co*-3HHx) with silk fibroin (SF) could improve the proliferation and osteogenic differentiation of human umbilical cord-derived mesenchymal stem cells (hUC-MSCs), which is a potential biomaterial for bone tissue engineering (Ang et al., 2020). Besides that, due to its elasticity, the P(3HB-*co*-3HHx) biomaterial could resist systemic pressure and support cell growth, making it a potential tissue-engineered blood vessel (Qu et al., 2006b). It is a potential biomaterial to replace synthetic blood vessels during surgeries and reduce the risk of infection.

In addition, P(3HB-*co*-3HHx) can be utilized as a drug delivery system in cancer treatment due to its biodegradability and nontoxicity. A combination of P(3HB-*co*-3HHx) with folic acid and loaded with etoposide could improve the medication delivery to tumors (Chang et al., 2014). Another application is P(3HB-*co*-3HHx) nanoparticles filled with an insulin phospholipid complex to make a form of insulin that has a long-acting release to treat diabetes. Peng and co-workers have made a thermosensitive hydrogel filled with P(3HB-*co*-3HHx) nanoparticles that can be injected and broken down through biological processes (Peng et al., 2013). This allows insulin to be released slowly and steadily. Due to the long-term basal insulin release by this combination of nanoparticle and hydrogel, which might reduce the frequency of injections of patients, this may assist not just the elderly with diabetes but also patients from other age groups.

## Conclusion

In conclusion, P(3HB-*co*-3HHx) copolymer is a type of PHA capable of replacing petroleum-based plastics in different applications like single-used plastics, medical devices, and packaging due to its similar properties and biodegradability. It has a superior biodegradation rate compared to petroleum-based plastics, hence leaving no or little pollution. P(3HB-*co*-3HHx) can be produced by PHA-producing microorganisms through various pathways like the PHA biosynthesis pathway involving *phaA*, *phaB*, and *phaC*, β-oxidation, and fatty acids *de novo* synthesis dependent on the carbon source fed during cultivation. However, the properties of P(3HB-*co*-3HHx) are dependent on its 3HHx molar composition. Hence, it is necessary to control the 3HHx molar composition of the P(3HB-*co*-3HHx) produced by using different PhaCs, engineering of PhaCs, and metabolic engineering of bacteria for P(3HB-*co*-3HHx) to be applied. With more knowledge and information regarding PhaCs and the metabolic pathway on PHA biosynthesis, P(3HB-*co*-3HHx) with the desired 3HHx molar composition can be produced, making them more suitable for their respective applications.
